# A Novel Electrochemical Sensor for Probing Doxepin Created on a Glassy Carbon Electrode Modified with Poly(4-Amino- benzoic Acid)/Multi-Walled Carbon Nanotubes Composite Film

**DOI:** 10.3390/s100908398

**Published:** 2010-09-08

**Authors:** Xiao-Li Xu, Fei Huang, Guo-Liang Zhou, Song Zhang, Ji-Lie Kong

**Affiliations:** Department of Chemistry & Center of Analysis and Measurement, Fudan University, Handan Road 220#, Shanghai 200433, China; E-Mails: 071022016@fudan.edu.cn (X.-L.X.); 041022013@fudan.edu.cn (F.H.); 072022057@fudan.edu.cn (G.-L.Z.)

**Keywords:** poly(4-aminobenzoic acid), multi-walled carbon nanotubes, nanocomposite, doxepin, detection

## Abstract

A novel electrochemical sensor for sensitive detection of doxepin was prepared, which was based on a glassy carbon electrode modified with poly(4-aminobenzoic acid)/multi-walled carbon nanotubes composite film [poly(4-ABA)/MWNTs/GCE]. The sensor was characterized by scanning electron microscopy and electrochemical methods. It was observed that poly(4-ABA)/MWNTs/GCE showed excellent preconcentration function and electrocatalytic activities towards doxepin. Under the selected conditions, the anodic peak current was linear to the logarithm of doxepin concentration in the range from 1.0 × 10^−9^ to 1.0 × 10^−6^ M, and the detection limit obtained was 1.0 × 10^−10^ M. The poly(4-ABA)/MWNTs/GCE was successfully applied in the measurement of doxepin in commercial pharmaceutical formulations, and the analytical accuracy was confirmed by comparison with a conventional ultraviolet spectrophotometry assay.

## 1. Introduction

**Figure 1.**
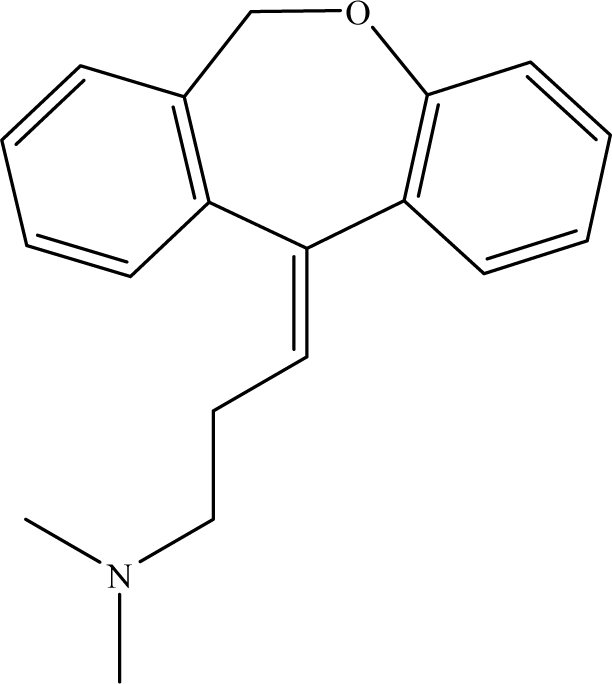
Molecular structure of doxepin.

Doxepin (Figure 1) has been widely used as an effective tricyclic antidepressant in the treatment of psychiatric disorders over the past decades [1,2]. Due to its importance, many analytical methods have been developed for its determination. Reported literature methods mainly focus on chromatographic techniques, such as reversed-phase liquid chromatography with ultraviolet detection [3], thin-layer chromatography-densitometry method [4], HPLC or LC-MS [5–8], and capillary zone electrophoresis [9], *etc.* Other methods are also available, including electrogenerated chemiluminescence [10], resonance light scattering [11], and the flow-injection techniques [12,13], *etc.* However, these methods are expensive, time-consuming and laborious.

Electrochemical methods provide an attractive alternative way to determine doxepin due to their simplicity, sensitivity, speed and low costs. There are only a few literature reports investigating the electrochemical properties of doxepin. Among the few examples, polarography [14], ion-selective membrane electrode [15] and boron-doped diamond electrode [16] were all used to study doxepin. Nevertheless, their applications in routine analysis of doxepin might be restricted due to toxicity of the dropping mercury electrode, sluggish response and unsatisfactory detection limit. Therefore, it is necessary to devise new determination procedures for doxepin.

Carbon nanotube (CNT)-modified electrodes have been successfully applied to study antidepressants [17], owing to the excellent conductivity and catalytic activity of CNTs. On the other hand, conductive polymers (CPs), with unique high stability and selectivity characteristics, good conductivity and reproducibility, more active sites and good homogeneity, have also been used to detect antidepressants [18–20]. These electrodes functionalized solely with CNTs or CPs presented excellent accumulation and electrocatalytic activities toward the targets.

Recently, electrodes functionalized with nanocomposite films of CNTs and CPs have been reported to determine the antidepressant trifluoperazine [21], for which a more sensitive signal response, faster response and lower detection limit could be obtained, compared with the CNT or CP film-modified electrodes. There is no report about the electrochemical determination of doxepin by CP/CNT composite film-modified electrodes.

In this paper, a new nanocomposite poly(4-ABA) and MWNT material was prepared by electrochemical polymerization. The poly(4-ABA)/MWNTs/GCE was characterized and applied to the detection of doxepin under optimum conditions. Analytical conditions, such as amount of MWNTs, electropolymerization conditions, pH and buffer solution, accumulation time and potential, were optimized.

## 2. Experimental Section

### 2.1. Reagents

Doxepin hydrochloride (C_19_H_21_NO·HCl) and its drug tablets were kindly supplied by Sine JiuFu Pharmaceutical Company (Shanghai, China). Doxepin stock solution (0.01 M) was prepared with double distilled water and stored at +4 °C in the dark. Buffer solution was 0.10 M phosphate buffer solution (PBS, pH 6.2). MWNTs (diameter 10–30 nm, length 0.5–40 μm) were supplied by Shenzhen Nanotech Port Co. Ltd. (Shenzhen, China). Before use, the received MWNTs were refluxed in a mixture of concentrated H_2_SO_4_ and HNO_3_ for about 6 h, then washed with double distilled water and dried under vacuum at room temperature. A suspension of MWNTs was prepared by dispersing MWNTs (2 mg) in *N,N*-dimethylformamide (2 mL) with the aid of ultrasonic agitation. 4-Aminobenzoic acid (4-NH_2_-C_6_H_4_-CO_2_H, 4-ABA) was purchased from Sinopharm Chemical Reagent Co., Ltd. 1.0 × 10^−4^ M 4-ABA solution was prepared with 0.10 M PBS (pH 7.5), and was stored at +4 °C in the dark. Before measurement, doxepin tablets were ground into powder, dissolved in water, filtered and diluted to a fixed volume. All the other chemicals were of analytical grade and used as received. Double distilled water was used exclusively in the experiments.

### 2.2. Apparatus

Electrochemical measurements were carried out on a CHI1030 multichannel voltammetric analyzer (ChenHua Instrument Company, Shanghai, China). The conventional three-electrode system was employed, including a platinum wire as counter electrode, a saturated calomel electrode (SCE) as reference electrode, and a bare GCE (Φ = 2.5 mm), poly(4-ABA)/GCE, MWNTs/GCE, and poly(4-ABA)/MWNTs/GCE as working electrode. Electrochemical impedance spectroscopy (EIS) measurements were performed on an EG&G PAR Model 273 A bipotentiostat (Princeton, NJ, USA) in conjunction with a lock-in amplifier. A 5-mV amplitude sine wave was applied to the electrode under potentiostatic control and the frequency range was from 0.05 Hz to 10 KHz. Scanning electron microscopy was performed on a Philips XL30 Microscope (Japan). UV-vis absorption spectra were obtained on an HP8453 ultraviolet-visible spectrophotometer (Agilent, Palo Alto, CA, USA).

### 2.3. Fabrication of the poly(4-ABA)/MWNTs/GCE

The nanocomposite film modified electrode was fabricated as follows: firstly, the bare GCE was polished sequentially with 0.3 and 0.05 μm Al_2_O_3_ slurry to form a mirror-like surface, then rinsed and ultrasonicated for 10 min in water, acetone, 1:1 HNO_3_ and water, respectively, and dried in air. The MWNTs/GCE was fabricated by dropping MWNT suspension (8 μL) on the cleaned GCE surface and evaporating the solvent in air. Secondly, the resulting MWNTs/GCE was immersed in 0.10 M PBS (pH 7.5) containing 1.0 × 10^−4^ M 4-ABA, and cyclic voltammetry was performed from −1.4 to +2.4 V for 10 cycles at a scan rate of 100 mV s^−1^ according to the literature [22]. Thus, the poly(4-ABA)/MWNTs/GCE was prepared. Before use, the modified electrode was continuously cycled from +0.3 to +1.2 V in a blank buffer solution of 0.10 M PBS (pH 6.2) until a stable cyclic voltammogram was obtained. All experiments were performed at about 25 °C.

## 3. Results and Discussion

### 3.1. Cyclic voltammograms of electropolymerization of 4-ABA and characterization of the modified GCE

**Figure 2.**
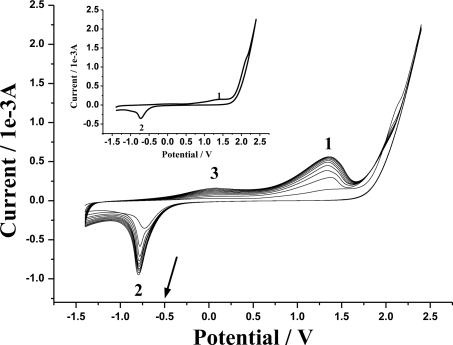
Repetitive cyclic voltammograms of 1.0 × 10^−4^ M 4-ABA at MWNTs/GCE. The insert is the first cycle of the cyclic voltammogram. Scan rate: 100 mV s^−1^; supporting electrolyte: 0.10 M PBS (pH 7.5).

The electropolymerization of 1.0 × 10^−4^ M 4-ABA in 0.10 M PBS (pH 7.5) at MWNTs/GCE is given in the repetitive cyclic voltammograms of Figure 2. As seen, in the first scan, an anodic peak 1 at about 1.37 V and a cathodic peak 2 at around −0.73 V appeared, respectively. From the second cycle on, another anodic peak 3 occurred at about 0.09 V. As the scans continued, the current of each peak increased, suggesting a continuous growth of the poly(4-ABA) film. Meanwhile, the current grew quickly in the initial five cycles, and then increased slowly until 10 scans. After the 10 scans, a thin adherent brown film could be found on the electrode surface. The electrochemical behavior of 4-ABA at MWNTs/GCE might be as follows [22,23]: 4-ABA was oxidized to free radical (peak 1) at first; the free radicals combined together rapidly to hydrazobenzoic acid; then hydrazobenzoic acid was oxidized to azobenzoic acid (peak 3), and azobenzoic acid reduced to hydrazobenzoic acid (peak 2).

**Figure 3.**
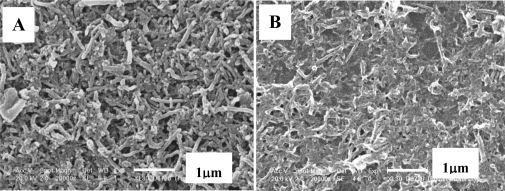
The surface images of MWNTs/GCE (A) and poly(4-ABA)/MWNTs/GCE (B) produced by scanning electron microscopy. Scale bar: 1 μm.

The surface morphology of MWNTs/GCE and poly(4-ABA)/MWNTs/GCE was investigated by scanning electron microscopy. As may be seen in Figure 3, MWNTs were well dispersed on the surface of the GCE (Figure 3A). After 4-ABA was polymerized in and on the MWNTs matrix, a stable, uniform, porous and three-dimensional film structure was formed (Figure 3B), which would presumably exhibit excellent interaction with doxepin molecules. It could be inferred that, the nanocomposite film might get more stable than the sole MWNTs film, as the thin layer of poly(4-ABA) could help fix the modified MWNTs and prevent them from leaving the electrode surface.

**Figure 4.**
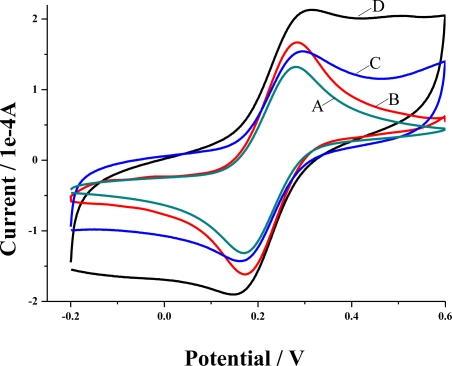
Cyclic voltammograms of 5 mM K_3_Fe(CN)_6_ at bare GCE (A); MWNTs/GCE (B); poly(4-ABA)/GCE (C) and poly(4-ABA)/MWNTs/GCE (D). Scan rate: 100 mV s^−1^.

Electrochemical responses of K_3_Fe(CN)_6_ at the bare GCE and the modified electrodes are shown in Figure 4. The largest peak current and background current at the poly(4-ABA)/MWNTs/GCE (Figure 4D) were obtained when compared with those at the MWNTs/GCE, poly(4-ABA)/GCE, and bare GCE, which indicates the composite effect of MWNTs and poly(4-ABA) might benefit the electron transfer of the electrochemical probe, Fe(CN)_6_^3−^. Moreover, the clear increase of peak current at the MWNTs/GCE (Figure 4B) compared with that of the bare GCE (Figure 4A), also suggests the prominent conductivity of MWNTs.

Meanwhile, the larger background current of the poly(4-ABA)/GCE (Figure 4C) than that of the bare GCE, implies the enhancement of the apparent area by the polymer. However, it is strange that a lower peak current at the poly(4-ABA)/GCE (about 11%) than that of the bare GCE was found, which might be ascribed to the electrostatic repulsion between polymer and probe. Herein, since the pK_a_ of 4-ABA is about 3.0 [24], carboxylic groups of the polymer are negatively charged due to the dissociation in neutral surroundings. Therefore, the poly(4-ABA) would repel Fe(CN)_6_^3−^ arriving at the electrode surface, and led to the observed current decrease.

**Figure 5.**
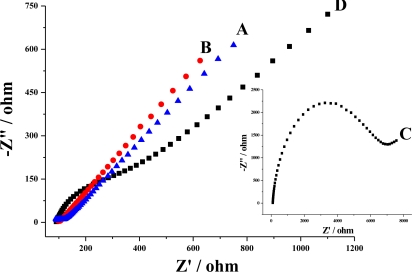
EIS plots of bare GCE (A), MWNTs/GCE (B), poly(4-ABA)/GCE (C), and poly(4-ABA)/MWNTs/GCE (D). Frequency used: 0.05 Hz to 10 KHz; solution: 5 mM K_3_Fe(CN)_6_/K_4_Fe(CN)_6_.

Figure 5 presents the EIS spectra obtained at the bare GCE (A), the MWNTs/GCE (B), poly(4-ABA)/GCE (C), and poly(4-ABA)/MWNTs/GCE (D). Plot B was a straight line, suggesting a fast electron transfer promoted by MWNTs. Plots A, C and D included a small semicircular part and a linear part, where the semicircular part at higher frequencies corresponded to the electron-transfer limited process, and the linear part at lower frequencies corresponded to the diffusion process.

The resistance of electron transfer for GCE, poly(4-ABA)/GCE and poly(4-ABA)/MWNTs/GCE are 48.8 Ω, 6,551 Ω, and 440.0 Ω, respectively. The clear increase of electron-transfer resistance at the poly(4-ABA)/GCE implies the modification of poly(4-ABA), which will block the electron-transfer of Fe(CN)_6_^3−/4−^ at the electrode due to the electrostatic repulsion between polymer and probe. This result is consistent with the previous cyclic voltammetry result. However, the electron-transfer resistance at poly(4-ABA)/MWNTs/GCE decreased obviously, which indicates the enhancement of conductivity by modification of the poly(4-ABA)/MWNTs composite film.

### 3.2. Electrochemical responses of the poly(4-ABA)/MWNTs/GCE

**Figure 6.**
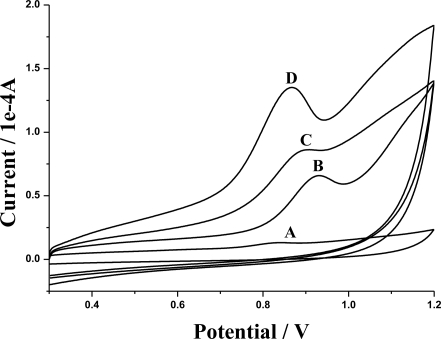
Cyclic voltammograms of 1.0 × 10^−5^ M doxepin at bare GCE (A), poly(4-ABA)/GCE (B), MWNTs/GCE (C), and poly(4-ABA)/MWNTs/GCE (D). Scan rate: 100 mV s^−1^; supporting electrolyte: 0.10 M PBS (pH 6.2); accumulation time: 60 s.

Figure 6 shows the electrochemical behavior of doxepin at the bare GCE (A), the poly(4-ABA)/GCE (B), the MWNTs/GCE (C), and the poly(4-ABA)/MWNTs/GCE (D). When the MWNTs or the poly(4-ABA) was introduced on the GCE, the current signal was amplified over 10-fold compared with that of the bare GCE, while the poly(4-ABA)/MWNTs/GCE exhibited the best anodic peak current among the four electrodes. The excellent sensitivity of the poly(4-ABA)/MWNTs/GCE could be ascribed to the presence of the nanocomposite film of MWNTs and poly(4-ABA), which might present excellent electrocatalytic activities and preconcentration towards doxepin. Furthermore, the oxidation potential of doxepin at the poly(4-ABA)/MWNTs/GCE was more negative compared with that of the MWNTs/GCE and the poly(4-ABA)/GCE, which also indicates the nice conductivity and the composite effect of the nanocomposite film. Additionally, no cathodic peak of doxepin was found on both bare GCE and modified electrodes, which suggests that the electrochemical oxidation of doxepin is irreversible.

### 3.3. Effect of scan rate

With scan rate increasing, the anodic peak of doxepin grew. It was found that the anodic peak current was linear to the scan rate in the range of 10–200 mV s^−1^, the regression equation was:

(1) $$I_{p}=1.010+0.657\text{v }\;(I_{p}\;(\mu A),\;v(\text{mV}\;s^{-1}),\;r=0.999)$$

This indicates the electrode process is adsorption-controlled. In additional, the anodic peak potential and the logarithm of scan rate also showed a linear relationship, following the equation:

(2) $$E_{p}=0.712+0.0299\;\text{lnv}\;(E_{p}\;(V),r=0.998)$$

According to the equation [25]:

(3) $$I_{p}=n^{2}F^{2}\text{vA}\Gamma_{0}^{*}/4\text{RT}=\text{nFQv/}4\text{RT}$$

the factor n was calculated to be 1.35, which indicates one electron is lost during the electrochemical oxidation. This result is consistent with the mechanism of doxepin electrooxidation proposed in the literature [10]. The electrooxidation of doxepin might take place at the nitrogen atom in the alkylamine, resulting in the formation of a cation radical, followed by deprotonation. In addition, according to the equation [26]:

(4) $$E_{p}=E^{\Phi }+[\text{RT}/(1-\alpha )\text{nF}]\text{ln}[{\text{RTk}}_{s}/(1-\alpha )\text{nF}]+[\text{RT}/(1-\alpha )\text{nF}]\text{lnv}$$

the charge-transfer coefficient α was calculated to be 0.15.

### 3.4. Optimization of the analytical conditions

#### 3.4.1. Influence of amount of MWNTs

As the volume of the MWNTs suspension was increased from 0 to 8 μL, the peak current response at the MWNTs/GCE was found to grow continuously. When the volume exceeded 8 μL, the response decreased. Apparently, the responses were closely related to the thickness of the MWNTs film. On the one hand, if the film was too thin, the number of adsorbed doxepin molecules was smaller, thus the response was smaller. On the other hand, when it was too thick, the film would become a little instable due to the desorption of MWNTs from the electrode surface, which led to the decrease of the peak current. Consequently, 8 μL MWNTs suspension was chosen for modification.

#### 3.4.2. Influence of electropolymerization conditions

The potential range for electropolymerization was very important for the preparation of the poly(4-ABA). The results showed that if the positive potential was lower than +1.5 V or the negative potential was higher than −0.8 V, the polymerization reaction hardly occurred. When the positive potential reached +1.8 V, the electropolymerization happened. It was found that, the polymer prepared between the potential window from −1.4 to +2.4 V exhibited much better electrochemical response to doxepin than the other ranges, thus the window was selected from −1.4 to +2.4 V.

According to the mechanism of the electropolymerization reaction of 4-ABA [23], a basic medium would be beneficial to the formation of the polymer. Thus, 0.10 M buffer solutions (pH 7.5) of PBS, Tris-HCl, Na_2_B_4_O_7_ and NH_3_-NH_4_Cl were initially examined. The result showed that the responses did not differ, indicating the medium had little effect on the electropolymerization. Furthermore, when the pH value of 0.10 M PBS varied from 7.0 to 9.0, the response at pH 7.5 was a little better than the other pH. Generally, 0.10 M PBS (pH 7.5) was selected as the polymerization medium for its mildness.

In addition, when the oxygen dissolved in the medium was removed before electropolymerization, the doxepin responses remained almost unchanged. This suggests oxygen is not involved in the polymerization reaction, which is consistent with the electropolymerization mechanism of 4-ABA, so it was generally not necessary to remove oxygen before electropolymerization.

Moreover, when the number of the polymerization cycles was increased from 0 to 25 scans, it was found that the current responses of doxepin grew until 10 scans, then they reached a plateau, and trended to decrease slightly for more scans. This suggests the modified GCE has the best electrochemical behavior for 10 scans. More scans would produce a denser film and the film became insulate. Therefore, 10 scans were selected as the best number of polymerization cycles.

#### 3.4.3. Influence of pH and buffer solution

**Figure 7.**
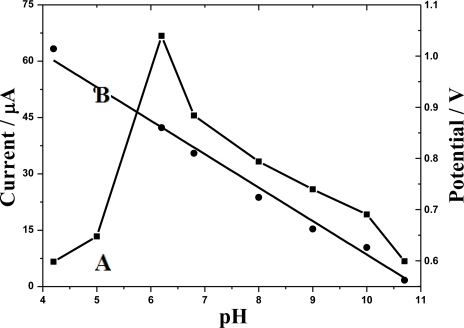
Influence of pH on anodic peak current (A) and peak potential (B) of doxepin. Supporting electrolyte: 0.10 M PBS. Other conditions as in Figure 6.

As shown in Figure 7A, within the range from pH 4.2 to 10.7, the anodic peak current of doxepin increased until it attained the maximum at pH 6.2, then it decreased. Additionally, the anodic peak potential shifted negatively with the pH increasing (Figure 7B), following the linear equation:

(5) $$E_{p}=1.276-0.0671\text{pH }(E_{p}\;(V),r=0.995)$$

This implies that H^+^ is involved in the oxidation of doxepin. According to literature [27], the pK_a_ of doxepin is 9.0. At much lower pHs, the oxidation generally became difficult due to the strong protonation and responses were lower. As the pH grew, the responses were enhanced due to the deprotonation. Nevertheless, neutral doxepin is hydrophobic [28], and as the pH increased further and exceeded the pK_a_, the solubility decreased gradually and drug precipitation occurred, which could affect the accumulation and caused the decrease of the responses. Therefore, pH 6.2 was chosen as the optimum pH value. Then several supporting electrolytes including PBS, Tris-HCl, Na_2_B_4_O_7_-HCl and HAc-NaAc were also tested. The results showed that the response obtained in PBS was a little better than that in other media. Besides, when the buffer concentration varied from 0.02 to 0.4 M, no obvious effect on the responses was observed. Hence, 0.10 M PBS (pH 6.2) was chosen as the buffer solution.

#### 3.4.4. Influence of accumulation time and potential

For a 1 × 10^−5^ M doxepin solution, the anodic peak currents increased as the accumulation time increased. After 60 s of preconcentration, the responses reached the maximum, and longer accumulation time exhibited no more current growth, which implies the accumulation reaches saturation. Therefore, 60 s was chosen as accumulation time. When the accumulation potential varied from −0.6 to +0.4 V, the peak current decreased continuously. However, compared with at open circuit, the effect of accumulation potential was negligible. Hence, open circuit accumulation was adopted.

### 3.5. Calibration curve

Under the optimal analytical conditions, the determination of doxepin at different concentrations was performed. The anodic peak current was linear to the logarithm of the concentration of doxepin from 1.0 × 10^−9^ to 1.0 × 10^−6^ M. The linear regression equation was expressed as:

(6) $$i_{p}=2.379+0.189\;\text{logC}\;(i_{p}\;(\mu A),\;C\;(M),r=0.997)$$

The detection limit for doxepin was estimated to be 1.0 × 10^−10^ M (S/N = 3), lower than that obtained by the boron-doped diamond electrode [16] and other chromatographic methods [7,29].

### 3.6. The reproducibility, regeneration, stability and selectivity of poly(4-ABA)/MWNTs/GCE

For eight parallel measurements of 1.0 × 10^−5^ M doxepin, the R.S.D. of the peak current was calculated to be 2.5%. The electrode could be easily regenerated by repetitive cycling in a blank solution of 0.10 M PBS (pH 6.2) for several times. The response of doxepin at the poly(4-ABA)/MWNTs/GCE could retain around 90% of the original response after seven days. This suggests the modified electrode has high stability.

The influence of foreign compounds was also tested at the poly(4-ABA)/MWNTs/GCE. It was found that several kinds of surfactants such as sodium dodecyl sulfate did not interfere, while Tween-20 and Triton X-100 could decrease the peak current. 50-fold Cu^2+^, Ag^+^, Fe^3+^, 100-fold epinephrine, vitamin C, antipyrine, norfloxacin, allopurinol, thiamine, glucose, phenylalanine, captopril, or hydroxyzine, had no obvious effect on the determination. This suggests the poly(4-ABA)/MWNTs/GCE has certain resistance to some interferences.

### 3.7. Applications

The poly(4-ABA)/MWNTs/GCE was applied to detect doxepin in drug tablets. The pretreatment and determination procedures for doxepin tablets were the same as described in Section 2. The analytical results were shown in Table 1, and the recovery was 98.0–100.4%. The doxepin content was calculated to be 24.7 mg per tablet (its declared content was 25 mg). In addition, the accuracy of this method was examined by comparison with the result from the UV assay proposed by the Chinese Pharmacopoeia (the UV absorbance of doxepin at 297 nm in methanol solution containing 0.01 M HCl was chosen as the quantitative criterion) [28]. By means of F-test and t-test, the calculated F-value (2.71) and t-value (0.56) were both smaller than theoretical values (5.05 and 2.23, respectively, at the 95% confidence level). It implies that the method proposed could be reliably used for routine analysis.

## 4. Conclusions

A sensitive electrochemical sensor for doxepin was prepared on GCE via modification of the nanocomposite film of the MWNTs and the poly(4-ABA). The excellent performance of the poly(4-ABA)/MWNTs/GCE could be ascribed to the effective preconcentration capacity and the excellent catalytic activities toward doxepin by the nanocomposite material. Thus, a sensitive detection method of doxepin due to the anodic peak was proposed. The as-prepared sensor with high sensitivity, speed, simplicity and low costs met the requirements of doxepin detection in drug tablets, and the procedure was proven to be reliable and could potentially be applied in clinical analysis of doxepin in physiological samples.

## 



## Figures and Tables

**Table 1.** Measurement results of doxepin in drug tablets (n = 6).

**Sample number**	**Added (μg/mL)**	**Expected (μg/mL)**	**Found (μg/mL)**	**R.S.D.(%)**	**Recovery (%)**	**UV method (μg/mL)**
0	0	1.00	0.99	2.5	99.0	0.98
1	1.00	2.00	1.96	2.9	98.0	1.99
2	2.00	3.00	2.97	2.1	99.0	2.98
3	3.00	4.00	3.94	3.3	98.5	4.02
4	4.00	5.00	5.02	1.7	100.4	4.99
