# Supplementary material for: A Novel Electrochemical Sensor for Probing Doxepin Created on a Glassy Carbon Electrode Modified with Poly(4-Amino- benzoic Acid)/Multi-Walled Carbon Nanotubes Composite Film
Source: Sensors (Basel). 2010 Sep 8;10(9):8398–410. doi: 10.3390/s100908398 (PMC3231211; doi:10.3390/s100908398)

**Abstract:** A novel electrochemical sensor for sensitive detection of doxepin (i.e. DXP) was prepared, which was based on glassy carbon electrode modified with poly(4-aminobenzoic acid)/multi-walled carbon nanotubes composite film (i.e. poly(4-ABA)/MWNTs/GCE). The sensor was characterized by SEM and electrochemical methods. The poly(4-ABA)/MWNTs/GCE showed excellent preconcentration function and electrocatalytic activities towards DXP. And it was successfully applied in the measurement of DXP in commercial pharmaceutical formulations, and the analytical accuracy was confirmed by comparison with conventional ultraviolet spectrophotometry.

**Keywords:** Poly(4-aminobenzoic acid); Multi-walled carbon nanotubes; Nanocomposite; Doxepin; Detection.

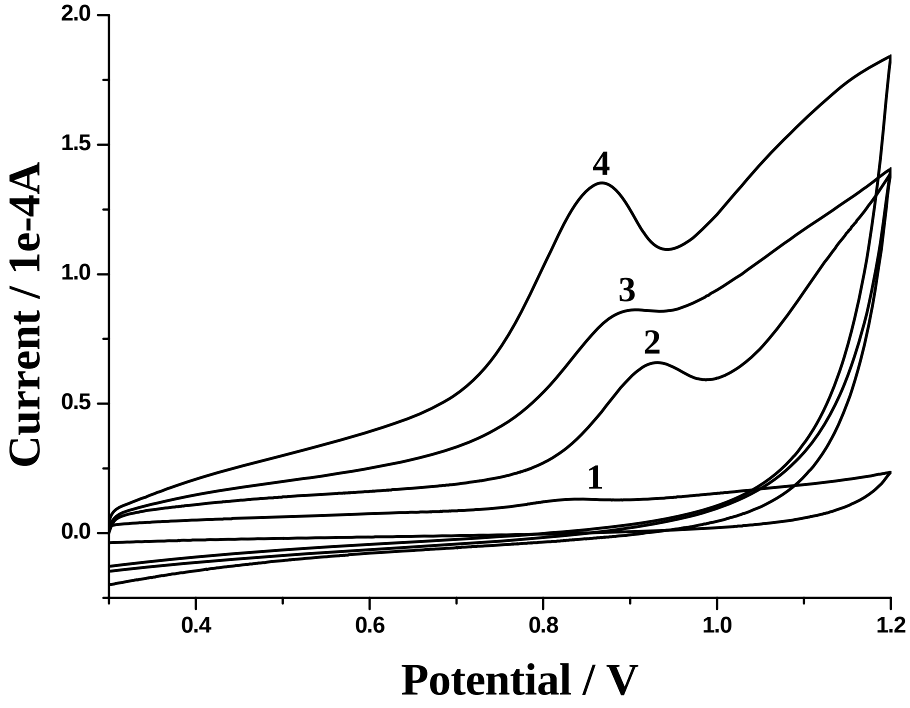

Supplement: Supplementary file 1 [file sensors-10-08398-s001.pdf]
